# Renal Inflammatory Myofibroblastic Tumor: A Case Report and Comprehensive Review of Literature

**DOI:** 10.4021/wjon287w

**Published:** 2011-04-09

**Authors:** Nora G. Lee, Mariam P. Alexander, Huihong Xu, David S. Wang

**Affiliations:** a Departments of Urology and Pathology, Boston University Medical Center, Boston, MA, USA

**Keywords:** Inflammatory myofibroblastic tumor, Inflammatory pseudotumor, Plasma cell granuloma

## Abstract

Inflammatory myofibroblastic tumor (IMT) is a rare benign tumor found in various organs with a sparse number of cases reported in the kidney. We report a case of IMT in a 48-year-old male who underwent laparoscopic partial nephrectomy for a 2.4 cm renal mass suspicious for renal cell carcinoma. Pathologic findings revealed spindle shaped cells in a myxoid background with lymphoid aggregates consistent with inflammatory myofibroblastic tumor.

## Introduction

Inflammatory myofibroblastic tumor (IMT) is a rare tumor that has been reported in multiple organs. Within the urogenital tract, the bladder is the most common site of IMT. However, there have been few cases reported in the kidney to date. In our review of the literature, there have been 38 previously reported case reports in the English-language medical literature between 1972 and 2010. IMT has also been termed inflammatory pseudotumor, plasma cell granuloma, and pseudosarcomatous fibromyxoid tumor. We report a case of IMT in a 48-year-old male at our institution found incidentally and will review the current literature.

## Case Report

**Figure 1:**
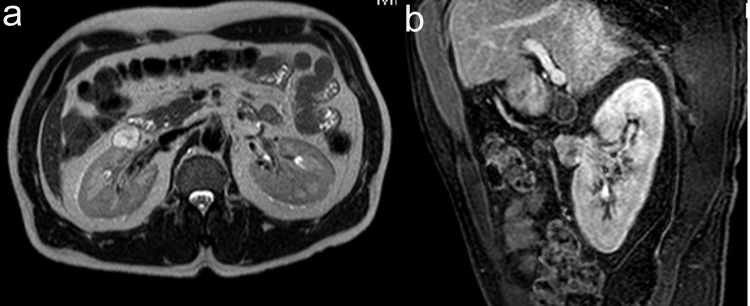
(a) 2.1 cm x 2.3 cm x 2.4 cm, T2 hyperintense, enhancing soft tissue renal mass anterior to interpole of right kidney. (b) Sagittal view of T1 weighted thrive sequence.

A 48-year-old male was referred to urology for an incidentally found right complex renal mass. The patient denied any urinary symptoms as well as gross hematuria. Urinalysis was also negative for microscopic hematuria. He has a past medical history of chronic Hepatitis B and Raynaud’s disease. The patient initially had an MRI study which revealed a 2.4 cm multi-septated cystic mass in the right kidney. The decision was made to place the patient on surveillance for this lesion. At 1-year follow-up, repeat MRI study revealed 2.4 cm T2 hyperintense, enhancing soft tissue renal mass concerning for renal cell carcinoma (Fig. 1). Given this change in appearance, patient underwent an uneventful right laparoscopic partial nephrectomy.

**Figure 2:**
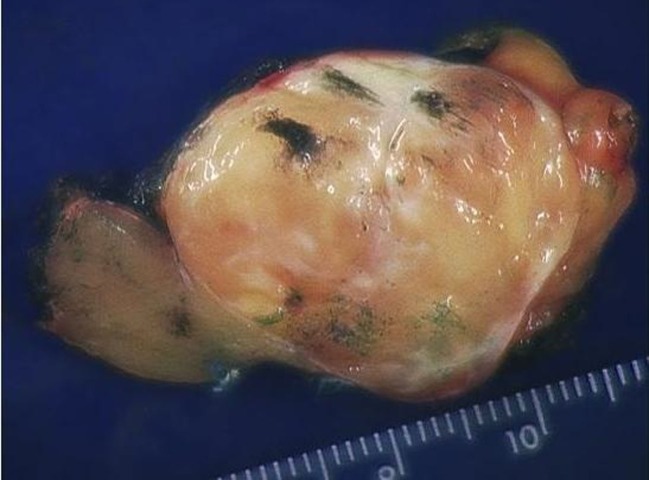
Gross specimen. Tumor is typically circumscribed, and unencapsulated with a tan, myxoid cut-surface.

**Figure 3:**
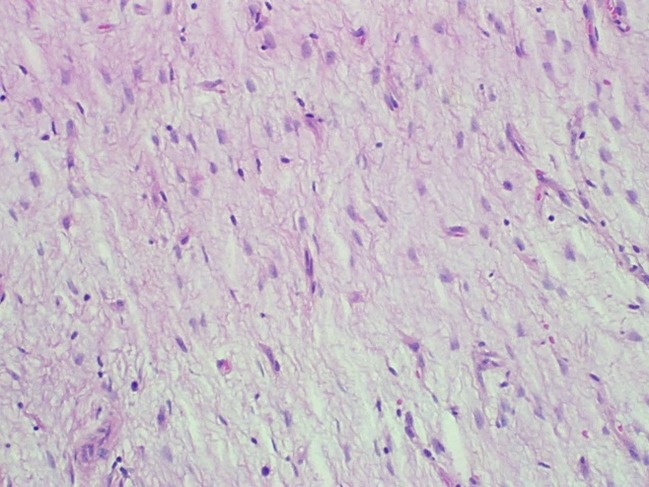
Hematoxylin and eosin staining (x 200). Loosely arranged spindle shaped fibroblasts devoid of nuclear pleomorphism and atypical mitotic figures with blood vessels in the background.

**Figure 4:**
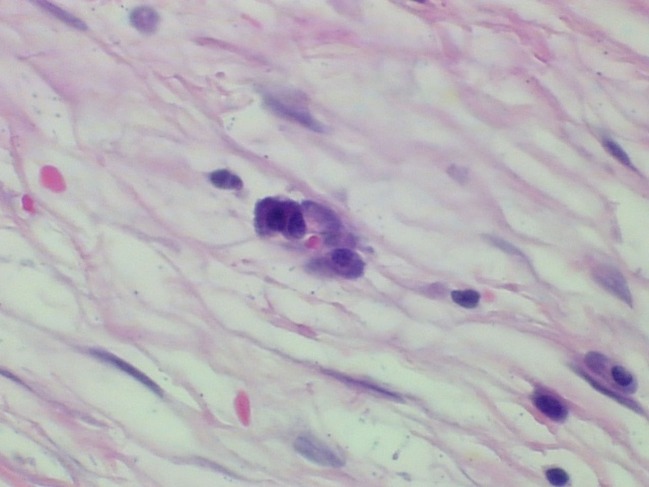
Hematoxylin and eosin staining (x 400). Plasma cells admixed with the spindle cells in a loose fibromyxoid background.

The specimen consisted of a mass weighing 22.8 grams and measuring 6 x 5 x 4.5 cm. The well-defined nodule itself measured 3.5 x 1.8 x 1.5 cm and was found to be gray/white in color with gelatinous consistency on gross examination (Fig. 2). Microscopically the specimen was composed of bland looking spindle shaped cells in a myxoid background and with lymphoid aggregates (Fig. 3, 4). Surgical margins were negative, and the diagnosis of inflammatory myofibroblastic tumor was made.

## Discussion

Inflammatory myofibroblastic tumor is a ubiquitous benign tumor which most commonly affects the lung and orbit [1]. This rare tumor has been described in most organs throughout the body. First described in the urogenital tract in the renal pelvis by Davides in 1972, IMT has additionally been identified in the urethra, prostate, ureter, epididymis and bladder, being the most common site in the genitourinary tract [2-5]. The kidney has been a rare site of IMT.

In our review of the English-language medical literature, there have been 38 other cases of IMT in the kidney that have been identified through a Pub-Med search of MEDLINE databases between 1972 and 2010. Predominantly these cases have been reported outside of the United States with over 5 cases reported within the U.S. Of the cases reviewed, 54% of the patients were male with ages ranging from 8 and 76 years (median 51 years) [6-9]. Four of these cases were reported in children [3]. The clinical presentation for IMT varies from an incidental finding (36%) to pain (38%), microscopic or gross hematuria (28%), and constitutional symptoms (23%) [6-9]. These lesions have been identified on imaging studies via US, CT and/or MRI. However given the heterogeneity of the appearance of these lesions, the preoperative exclusion of malignancy in these lesions is extremely difficult. The differential diagnosis of a renal IMT includes renal cell carcinoma, transitional cell carcinoma, malignant lymphoma, xanthogranulomatous pyelonephritis, angiomyolipoma with minimal fat, rhabdomyosarcoma in adults and Wilm’s tumor in children. In the cases reviewed, 85% (33/39) underwent unilateral partial or radical nephrectomy given the risk of malignancy in these lesions. There were no recurrences seen in the cases which reported follow-up [6-9]. The remaining 15% was treated with corticosteroid therapy in bilateral renal lesions for biopsy proven IMT which resulted in resolution of clinical findings [6, 10-12].

Histologically IMT is characterized by spindle-shaped cells that are mixed with a chronic inflammatory component consisting of plasma cells, lymphocytes, and occasionally histiocytes. Coffin et al. have described three histologic patterns: a myxoid and vascular pattern with inflammatory infiltrate, compact spindle cells proliferation, and hypocellular fibrous pattern [1]. Immunohistochemical studies support the myofibroblastic nature with consistent expression of vimentin and smooth muscle actin with variable positivity for HHF-35, cytokeratins, and CD68. Electron microscopy reveals spinal cells with abundant endoplasmic reticulum, pinocytotic vesicles, basal lamina, and extracellular collagen [1].

The etiology of IMT is unknown; however initially it was thought that these tumors represented a reactive inflammatory process, hence the name “pseudotumor”. The consistent pathologic features of mixed inflammatory infiltrate, vascularity resembling granulation tissue, lack of mitosis and polymorphism support this reactive cause theory along with its response to corticosteroids. This inflammatory reaction may be secondary to surgery, trauma, or infection. IMT has been associated with a variety of infectious agents including actinomyces and nocardiae in hepatic and pulmonary pseudotumors, mycoplasma in pulmonary lesions, and Epstein-Barr virus in splenic and nodal tumors [13]. Alternatively, it has been postulated that IMT has an autoimmune origin given its association with various disease processes noted in several case reports. It has been noted that a patient with IMT of the submandibular gland was also found to have polyclonal hypergammaglobulemia, high antinuclear antibody titers, and a positive antithyroid test without any symptoms of any specific systemic autoimmune disease [14]. In our case, our patient also had a history of Raynaud’s disease which may represent an autoimmune origin. On the contrary, others have felt that IMT is regarded as a neoplastic process with low malignant potential but can be locally aggressive [1]. Recent studies have found cytogenetic clonal abnormalities including aberrations of the anaplastic lymphoma kinase (ALK) gene at 2p23 supporting a neoplastic etiology [15].

Given the rarity of this tumor, no prospective studies have been performed on inflammatory myofibroblastic tumors of the kidney. As more of these tumors are identified, additional knowledge will be available to better understand the etiology and pathogenesis of this tumor.

### Conclusion

Renal inflammatory myofibroblastic tumor is a rare tumor which has been identified in less than 40 cases within the English-language medical literature. Given its often indistinguishable appearance on imaging from malignant renal cell carcinoma, nephrectomy is often the treatment of choice. Fortunately with the advent of partial nephrectomies and its more widespread application, the decision for surgical treatment of these benign tumors has become less considerable. Ultimately, the outcome for these patients is quite favorable.
